# Amino Acid Insertion Reveals a Necessary Three-Helical Intermediate in the Folding Pathway of the Colicin E7 Immunity Protein Im7

**DOI:** 10.1016/j.jmb.2009.07.085

**Published:** 2009-10-02

**Authors:** Stuart E. Knowling, Angelo Miguel Figueiredo, Sara B.-M. Whittaker, Geoffrey R. Moore, Sheena E. Radford

**Affiliations:** 1 Astbury Centre for Structural Molecular Biology, University of Leeds, Leeds LS2 9JT, UK; 2 Institute of Molecular and Cellular Biology, University of Leeds, Leeds LS2 9JT, UK; 3 Centre for Molecular and Structural Biochemistry, School of Chemical Sciences and Pharmacy, University of East Anglia, Norwich NR4 7TJ, UK; 4 School of Cancer Sciences, University of Birmingham, Vincent Drive, Edgbaston, Birmingham B15 2TT, UK

**Keywords:** Im7, inhibitor protein for colicin E7 providing immunity to cells producing the colicin E7, Im7H3M3, Im7 variant containing engineered helix III, K_XY_, the equilibrium constant between X and Y, k_XY_, the rate constant for the conversion of X to Y, M_XY_, the denaturant-dependence of the free energy between X and Y, m_XY_, the denaturant-dependence of the natural logarithm of the rate constant k_XY_, U, unfolded state, I, intermediate state, TS, transition state, N, native state, β_X_, Beta-Tanford values of species X, TFE, 2,2,2-trifluoroethanol, CD, circular dichroism, polyalanine, protein folding, ϕ-value analysis, helix design, intermediate

## Abstract

The small (87-residue) α-helical protein Im7 (an inhibitor protein for colicin E7 that provides immunity to cells producing colicin E7) folds *via* a three-state mechanism involving an on-pathway intermediate. This kinetic intermediate contains three of four native helices that are oriented in a non-native manner so as to minimise exposed hydrophobic surface area at this point in folding. The short (6-residue) helix III has been shown to be unstructured in the intermediate ensemble and does not dock onto the developing hydrophobic core until after the rate-limiting transition state has been traversed. After helix III has docked, it adopts an α-helical secondary structure, and the side chains of residues within this region provide contacts that are crucial to native-state stability. In order to probe further the role of helix III in the folding mechanism of Im7, we created a variant that contains an eight-amino-acid polyalanine-like helix stabilised by a Glu-Arg salt bridge and an Asn-Pro-Gly capping motif, juxtaposed C-terminal to the natural 6-residue helix III. The effect of this insertion on the structure of the native protein and its folding mechanism were studied using NMR and ϕ-value analysis, respectively. The results reveal a robust native structure that is not perturbed by the presence of the extended helix III. Mutational analysis performed to probe the folding mechanism of the redesigned protein revealed a conserved mechanism involving the canonical three-helical intermediate. The results suggest that folding *via* a three-helical species stabilised by both native and non-native interactions is an essential feature of Im7 folding, independent of the helical propensity of helix III.

## Introduction

**Fig. 1:**
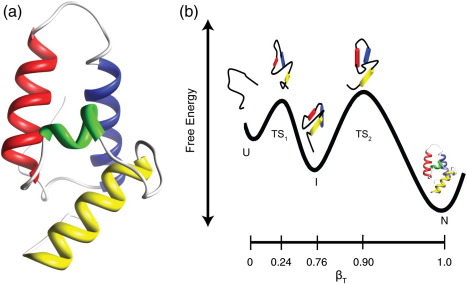
(a) Structure of wild-type Im7, drawn from the coordinates of 1AYI.^10^ Helices are coloured as follows: helix I, blue; helix II, red; helix III, green; helix IV, yellow. The figure was drawn using Chimera.^34^ (b) Cartoon representation of the folding mechanism of Im7. The on-pathway intermediate forms on the sub-millisecond timescale. How helices I, II and IV dock in the intermediate and transition state ensembles is shown schematically but is based on restrained molecular dynamics simulations of these species using Φ-values or hydrogen exchange protection factors as restraints.^1,43^ The helical structure in the unfolded state in the absence of denaturant is not known, although it is expected to be low, as predicted using AGADIR.^12^

Previous studies have shown that the small four-helical protein Im7 (an inhibitor protein for colicin E7 that provides immunity to cells producing colicin E7; Fig. 1a) folds *via* an unusually rugged folding landscape involving the population of an on-pathway hyperfluorescent intermediate.^1,2^ This species contains a core of hydrophobic residues that is ∼ 20% expanded compared with the native state (Fig. 1b) and is stabilised by both native and non-native interactions.^1–4^ The intermediate ensemble lacks a structured helix III, but contains helices I, II and IV, which are oriented in a non-native manner so as to minimise the exposed hydrophobic surface area that would result from a native-like helical organisation in the absence of helix III. The low ϕ-values for point mutations in the sequence that forms the native helix III in both the intermediate-state ensemble and the subsequent rate-limiting transition-state ensemble suggest that helix III only docks onto the developing protein core subsequent to crossing the rate-limiting transition-state barrier as the native structure develops (Fig. 1b). More specifically, residues Leu53 and Ile54, which are in helix III of the native structure and form an integral part of the hydrophobic core of the native protein, appear to play little or no role in stabilising the intermediate state.^1,5^ By contrast, either or both of the natively solvent-exposed or partially exposed residues Tyr55 and Tyr56 in helix III, which are essential for the inhibitory action of nuclease E colicin immunity proteins, have been suggested to form non-native interactions during folding, helping to anchor the stretch of residues that will ultimately form helix III onto the three-helix intermediate.^2,6–8^

Although much less stable than the intermediate in Im7 folding, an intermediate has been shown to form transiently during the folding of the Im7 homologue Im9.^9^ These data suggest that formation of a three-helical intermediate is a ubiquitous feature of the folding mechanism of immunity proteins, with specific side-chain–side-chain interactions in different proteins stabilising the folding intermediates to different extents. This raises the intriguing question of whether the short nature and low helical propensity of the sequence comprising the native helix III, which is highly conserved (83%) across the family of immunity proteins,^10^ are responsible for the development of the three-helical intermediate, or whether other features of the protein sequence dictate three-state folding. In order to address this question, we describe here a series of experiments in which the sequence of helix III was redesigned to increase its length and helical propensity through the insertion of an eight amino acid polyalanine-like helix without disruption of the native Im7 structure. The resulting newly extended helix III is predicted to have a length and a helical propensity that exceed those of helices I, II and IV. Here, we describe the design of this variant Im7 and the determination of its structure using NMR spectroscopy. In parallel, by analysis of the folding mechanism using ϕ-value analysis, we provide evidence that suggests an obligate requirement for folding *via* a three-helical intermediate, irrespective of the length and helical propensity of helix III.

## Results

### Construction of a variant of Im7 with a highly helical helix III

**Fig. 2:**
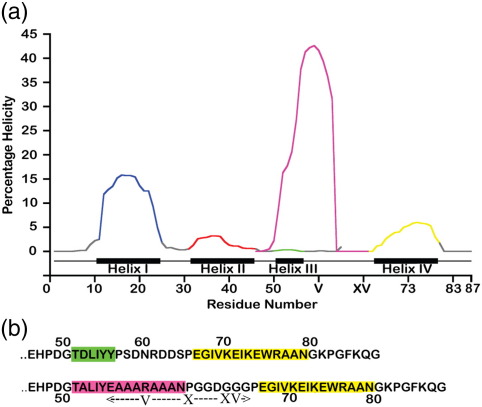
(a) Prediction of the helical propensities of the four helices in wild-type Im7 and the Im7H3M3 variant using the prediction algorithm AGADIR.^12^ Residues in helices are highlighted in accordance with Fig. 1a. Residues in unstructured regions are shown in grey. The helical propensity of wild-type Im7 helix III is shown in green, while that of Im7H3M3 is shown in purple. (b) Primary sequence of residues in helices III and IV and their sequences in Im7 (top) and Im7H3M3 (bottom). Helix III (green) and helix IV (yellow) in wild-type Im7 are shown. Residues predicted to occupy helix III in Im7H3M3 are shown in purple.

On average, α-helices in native proteins contain 12 residues.^11^ Helices I, II and IV of Im7, containing 13, 14 and 14 residues, respectively, conform to this view (Table 1). These three helices have average helical propensities of ∼ 8%, 2% and 3%, respectively, as predicted by AGADIR^12^ (Table 1 and Fig. 2a). By contrast, helix III of Im7 has only six residues and no significant helical propensity (Fig. 2a). Of the six residues that comprise helix III (Thr51, Asp52, Leu53, Ile54, Tyr55 and Tyr56), Asp52 and Tyr56 are highly solvent exposed, while the remaining residues are either totally buried or partially buried in native Im7, leading to stabilisation of the helical structure of this sequence as the native helix III. In order to increase the helical propensity of helix III, we initially considered substituting both Asp52 and Tyr56 with Ala. According to predictions from AGADIR,^12^ however, these substitutions do not significantly increase the predicted helical propensity of helix III. To increase the helical propensity of helix III further without perturbing the structure of the native protein, especially its native hydrophobic core, we found it necessary to extend the length of the helix. It has been shown that polyalanine sequences have a high helical propensity, even in the absence of tertiary contacts.^13,14^ Concatenation of such a sequence with that of the native helix III was thus considered to be a possible route towards the redesign of helix III to a length and a helical propensity commensurate with those of helices I, II and IV.

Three variants of helix III were designed and tested. First, the natural sequence of helix III (TDLIYY) was altered to TALIYA (substituting the exposed Asp52 and Tyr56 with Ala) without an increase in predicted helicity. This sequence was then expanded to contain, in total, an additional eight-residue helical segment based on the polyalanine TALIYAAAAAAAA, which increased the average predicted helicity of the sequence to 7%. Incorporation of this sequence into Im7, creating variant Im7H3M1, did not significantly alter the stability of the native protein compared with that of wild-type Im7 (Δ*G*_UN_^o^ = − 24.1 ± 0.7 kJ mol^−^ ^1^ and Δ*G*_UN_^o^ = − 25.6 ± 0.32 kJ mol^−^ ^1^, respectively; data not shown).^15^ The addition of residues Asn-Pro-Gly at the C-terminus of the newly incorporated sequence, which is known to form an efficient C-capping motif,^16^ to give an insert sequence of TALIYAAAAAAAANP increased the average predicated helical propensity to 10%, again without significantly altering the stability of the resulting protein Im7H3M2, which had a Δ*G*_UN_^o^ of − 22.5 ± 0.67 kJ mol^−^ ^1^ (data not shown).^15^

In order to further stabilise the engineered helix, we introduced a potential salt bridge into the inserted region as another contributor to α-helix stability. Such side-chain–side-chain interactions potentially contribute between 0.4 and 2.0 kJ mol^−^ ^1^ to the stability of a protein.^17–19^ As noted above, Asp52 and Tyr56 of Im7 are solvent exposed,^10^ and if the inserted region adopts the same helical structure, the residues at position I (originally Tyr56) and position V of the insert (Fig. 2b) are predicted to be solvent exposed. Therefore, the alanine residues at insert positions I and V were substituted with Glu and Arg, respectively to create Im7H3M3. As summarised in Fig. 2b, the redesigned helix III in Im7H3M3 is expected to contain a C-terminal-capped polyalanine helix with a salt bridge located towards its centre. Together, these stabilising features are predicted to increase the average helical propensity of helix III (over all residues) to 14% (Fig. 2a and Table 1). The variant Im7H3M3 thus contains a sequence for helix III that has the largest predicted helical propensity of all four helices in the protein, while maintaining the key hydrophobic side chains of Leu53 and Ile54 in the native hydrophobic core. In addition to the inserted sequence, Im7H3M3 differs from Im7 in the sequence linking helices III and IV. The wild-type interhelix connection (PSDNRDDS) was substituted in Im7H3M3 with a linker (GGDGGGP) that is expected to have a flexibility greater than that of the native interhelix linker to allow the possibility of the new polyalanine-rich helix III being accommodated into the structure without perturbing the docking of helix IV in the variant Im7 protein. In addition to the five Gly residues in the inserted linker, aspartic acid was incorporated to aid solubility.

Im7H3M3 folds into a helical structure, as judged by far-UV CD (data not shown), with a Δ*G*_UN_^o^ of − 23.3 ± 0.4 kJ mol^−^ ^1^, determined by equilibrium denaturation and chevron analysis (see the text below), similar to the properties of wild-type Im7 (Table 2). This variant was therefore used to study the importance of the length and helical propensity of helix III in the folding mechanism of Im7.

### CD studies of peptide fragments of Im7 and Im7H3M3

**Fig. 3:**
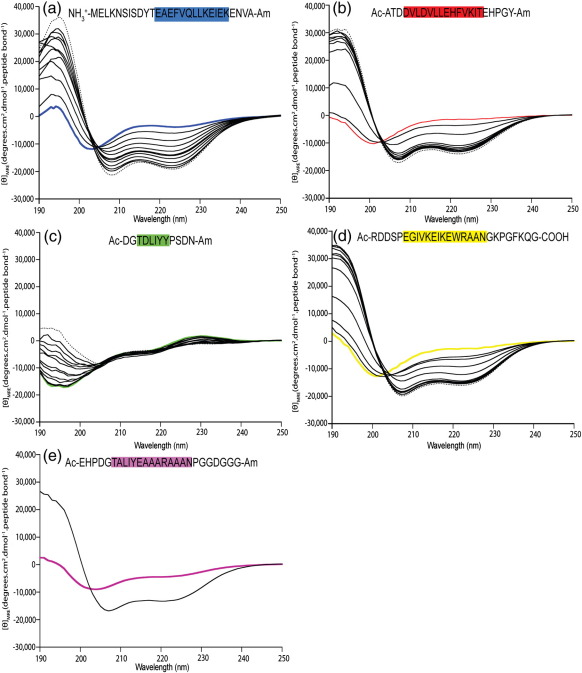
TFE titration curve of peptides corresponding to different Im7 helices. Helix I (a), helix II (b), helix III (c) and helix IV (d). The positions of native helices are shown in blue, red, green and yellow, respectively, in each plot. Data were acquired at 10 °C in 50 mM sodium phosphate buffer (pH 7.0). In each case, the spectrum in the absence of TFE is coloured, the spectra in 40% (vol/vol) TFE are shown as dotted lines and the spectra at intermediate concentrations of TFE are shown in continuous black lines. The spectra of helix III in Im7H3M3 are shown in 0% (vol/vol) TFE (purple) and 40% (vol/vol) TFE (black) (e).

In order to ascertain whether the polyalanine-rich sequence of the helix III insert in Im7H3M3 has increased its helical content, as predicted by its enhanced helical propensity, we synthesised a peptide equivalent to this sequence and measured its helical content in aqueous solution and in the presence of 2,2,2-trifluoroethanol (TFE) using far-UV CD.^20^ In parallel, peptides equivalent to the sequences of helices I, II and IV and of the natural helix III were also synthesised and studied under identical conditions (Fig. 3).

The far-UV CD spectrum of the Im7H3M3 helix III peptide in 0% vol/vol TFE shows a signal typical of an α-helix with minima at 208 and 222 nm (Fig. 3e), indicating that the sequence adopts a helical structure in solution,^21^ consistent with its design. The addition of 40% (vol/vol) TFE stabilises the helix, as observed for other polyalanine helices,^20^ although propagation of helicity throughout the peptide is presumably blocked by the presence of the proline residue towards its C-terminus.^22^ In aqueous solution (0% vol/vol TFE), the ellipticity of helix III peptide from Im7H3M3 results in an average helical content of 16%, determined from the magnitude of the signal at 222 nm^21^ (see Materials and Methods), that is close to the average predicted helical propensity of this sequence (∼ 14%) (Table 1).^12,23,24^ As shown in Fig. 2a and Table 1, peptides with sequences equivalent to those of helices I, II and IV were predicted to have average helix propensities of ∼ 8%, 2% and 3%, respectively. Each peptide, as measured by TFE titration, underwent a two-state transition into helical structure, as indicated by the presence of an isodichroic point in each titration series at 203 nm (also see Materials and Methods) (Fig. 3a, b and d). These experiments suggested average helicities of the peptides, determined from the magnitude of the signal at 222 nm (see Materials and Methods), that are equivalent to those of helices I, II and IV (14%, 10% and 11%, respectively—values significantly greater than those predicted by AGADIR) (Table 1). The peptide with the sequence of the natural helix III (Fig. 3c), by contrast, showed very little helical content in the absence of TFE and no change upon addition of TFE, indicating that this small 6-residue sequence requires tertiary structure formation for it to stabilise in helical form in native Im7.

### Structure of Im7H3M3 and side-chain interactions

**Fig. 4:**
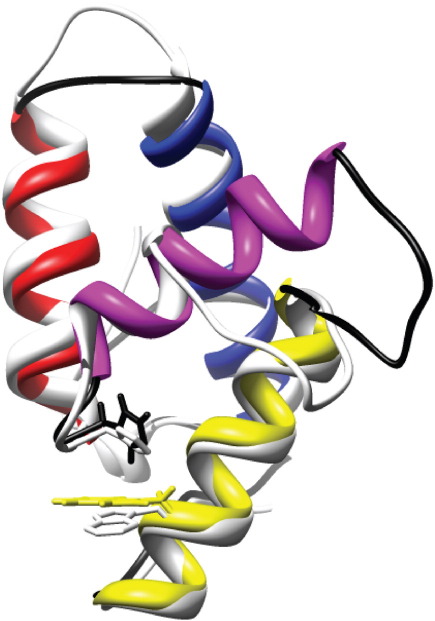
Representation of the structure of Im7H3M3, drawn from the coordinates of 2K0D. The structure of wild-type Im7 (1AYI)^10^ is shown in light grey for comparison. Helices are coloured as follows: helix I, blue; helix II, red; helix III, purple; helix IV, yellow. The positions of His47 (black) and Trp75 (yellow) in Im7H3M3 are shown, with their equivalents in Im7 shown in grey. This figure was drawn using Chimera.^42^

The solution structure of Im7H3M3 was determined by heteronuclear NMR spectroscopy (see Materials and Methods). The results revealed a structure that is remarkably similar to that of wild-type Im7, with helices I, II and IV adopting native-like conformations, as reflected by their lengths, orientations and side-chain arrangements (Fig. 4). As predicted, helix III has been extended by the polyalanine insert, entirely consistent with the redesign strategy. Also as predicted, the NMR structure indicates that both the C-cap motif and the Glu-Arg salt bridge in helix III are formed; the dihedral angles are consistent with a C-cap, while the average distance between the side-chain N atoms in Arg V and the carboxylate O atom in Glu I is ∼ 3 Å, consistent with the presence of a salt bridge. While a full description of the structure and dynamics of the Im7H3M3 variant will be given elsewhere (manuscript in preparation), it is noteworthy at this point to highlight the slightly different orientation of Trp75 in the two structures (Fig. 4), since this residue forms the fluorophore that is used as a probe for folding and stability in the studies described below. The indole ring remains stacked against the side-chain imidazole of His47 in Im7H3M3, a characteristic feature of immunity proteins that gives rise to the low fluorescence intensity of Trp75 in the native structure.^25^

### Kinetic analysis of Im7H3M3 folding

**Fig. 5:**
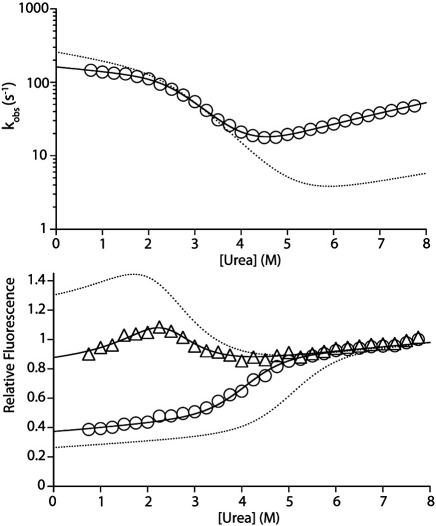
Denaturant dependence of the folding/unfolding rate constants of Im7H3M3, together with the initial and final fluorescence signals for folding. Top: The observed rate constants (circles) for the folding/unfolding of Im7H3M3 plotted as a function of urea concentration. The data were obtained at pH 7.0, 10 °C and 0.4 M Na_2_SO_4_. The continuous line shows the best fit of the data to a model describing a three-state transition. The broken line shows the data for wild-type Im7. Bottom: The initial (triangles) and final (circles) fluorescence signals for the folding of Im7H3M3. The continuous lines show the initial and final fluorescence signals fitted simultaneously with the rate constants. The broken line represents the best fit of the data obtained for wild-type Im7.

To determine whether the redesign of the helix III insert has affected the folding mechanism of Im7, we analysed the folding and unfolding kinetics of Im7H3M3 using stopped-flow fluorescence (10 °C, pH 7.0 and 0.4 M Na_2_SO_4_) (see Materials and Methods). The resulting chevron plot (Fig. 5) shows that the sequence differences between Im7H3M3 and wild-type Im7 destabilise the native state slightly (ΔΔ*G*_UN_^o^ ∼ 2.3 kJ mol^−^ ^1^), with a corresponding increase (4-fold) in the rate constant of unfolding (*k*_NI_) relative to that of wild-type Im7 (Table 2). Remarkably, the folding branch of the chevron plot of Im7H3M3 is unperturbed compared with that of wild-type Im7, indicating that Im7H3M3 folds *via* a three-state mechanism involving a hyperfluorescent intermediate (Fig. 5). Quantitative analysis of the data revealed that the stability of the intermediate in Im7H3M3 is increased relative to the stability of the intermediate that is transiently populated in wild-type Im7 (ΔΔ*G*_UI_^o^ ∼ 2.4 kJ mol^−^ ^1^) (Table 2). The Im7H3M3 variant exhibits an *m*-value for the formation of the intermediate (*M*_UI_; *M*_*XY*_ represents the denaturant dependence of the free energy between *X* and *Y*) that is approximately 15% larger than that of wild-type Im7 (Table 2), consistent with the increased length of the polypeptide chain and near-identical native structures of the two proteins. Accordingly, the *m*-value for folding from the unfolded protein to the native state (*M*_UN_) for Im7H3M3 (5.8 ± 0.2 kJ mol^−^ ^1^ M^−^ ^1^) is increased by 10% relative to that observed for wild-type Im7 (5.3 ± 0.1 kJ mol^−^ ^1^ M^−^ ^1^). Interestingly, although remaining hyperfluorescent, the fluorescence intensity of the intermediate formed during the folding of Im7H3M3 is reduced compared with the intermediate formed in the folding of wild-type Im7. Further experiments showed that this results, in part, from the substitution of Tyr56 with Ala, which must subtly alter the environment of Trp75 in the intermediate ensemble such that its fluorescence quantum yield is reduced.^15^

The small differences observed in the folding behaviour of Im7H3M3 compared with that of wild-type Im7 is surprising given that 9 native residues have been removed and 16 non-native residues have been inserted, including a region predicted by peptide studies to be significantly helical even in the denatured state (Fig. 3e). It has previously been shown that the insertion of only 4 alanine residues into a loop region after helix VIII of T4 lysozyme destabilises its native state by ∼ 25 kJ mol^−^ ^1^_,_ with a similar result being observed for the insertion of 3 alanine residues into a loop region after helix III in the same protein.^26^ T4 lysozyme is approximately twice the size of Im7. It is striking, therefore, that the smaller Im7 tolerates the insertion without substantial perturbation of native-state stability (ΔΔ*G*_UN_^o^ = 2.3 kJ mol^−^ ^1^) (Table 2). One possible explanation for the tolerance of Im7 for the polyalanine insertion is that the glycine linker allows the native structure to accommodate the extended helix III without causing strain on helix IV. Of particular interest is the stabilisation of the intermediate ensemble in Im7H3M3 compared with the intermediate of wild-type Im7 (ΔΔ*G*_UI_^o^ = 2.5 kJ mol^−^ ^1^). Work performed on an intermediate ensemble of Im7 trapped at equilibrium by the mutation of Leu53 and Ile54 to alanine^5^ shows that this variant displays an increased exposed hydrophobic surface area relative to the native state. Therefore, it is plausible that the polyalanine helix of Im7H3M3 helps protect this hydrophobic surface area from solvent in the intermediate ensemble, as was observed for the insertion variant L164-[AAAA] of T4 lysozyme.^26^

### ϕ-Value analysis

**Fig. 6:**
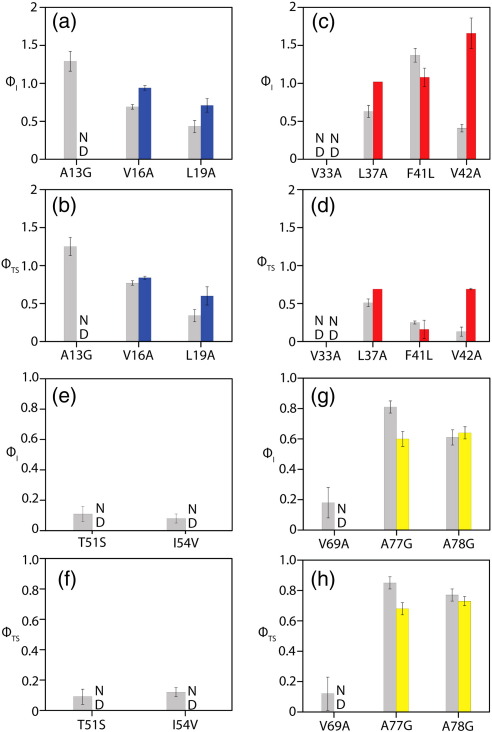
Comparison of ϕ_I_ and ϕ_TS_ for Im7 (grey) and Im7H3M3 (coloured). (a and b) Helix I, (c and d) helix II, (e and f) helix III and (g and h) helix IV. ND (not determined): residues that display ΔΔ*G*_UN_^o^ < 2.5 kJ mol^−^ ^1^ precluding the determination of a ϕ-value.^27^

In order to determine in more detail the effect of the sequence differences between Im7 and Im7H3M3 on the folding mechanism of Im7, we created a number of variants of Im7H3M3 (Table 3). Hydrophobic substitutions were made at residues in the core of the protein that have previously been shown to play a key role in the folding mechanism of Im7,^1,2^ so that the formation of tertiary structure contacts during folding could be investigated. In addition, to probe the formation of secondary structure, we made a series of alanine-to-glycine substitutions at solvent-exposed sites that included residues in all four helices. The folding and unfolding kinetics of all variants were then assessed using stopped-flow fluorescence, and the results were collated as a series of ϕ-values. Altogether, 24 variants that span the squence of Im7H3M3 were studied. Hydrophobic substitutions made in the centre of helix I (V16A and L19A) show that these residues, which are buried in the native state, stabilise the intermediate-state and transition-state ensembles in both wild-type Im7 and Im7H3M3, resulting in ϕ_I_ and ϕ_TS_ values of > 0.6 for both Im7HM3 and wild-type Im7 (Fig. 6a and b and Table 4). These side chains, therefore, are buried early in the folding of both wild-type Im7 and Im7H3M3.

When considered as a whole, the variants of Im7H3M3 in helix II that were examined by mutational analysis also showed properties similar to those obtained for Im7 (Fig. 6c and d). L37A, F41L and V42A show high ϕ_I_ values in Im7H3M3 (1.02 ± 0.0, 1.08 ± 0.12 and 1.66 ± 0.2, respectively) that are reduced in the transition-state ensemble (0.69 ± 0.00, 0.16 ± 0.12 and 0.69 ± 0.01, respectively). Despite the similarity in ϕ-values for residues in helices I and II, some subtle structural reorganisation of the intermediate ensemble in Im7H3M3 may have occurred relative to that of Im7, however, as reflected in the increased ϕ-values of V16A, L19A (Fig. 6a and b) L37A and V42A in Im7H3M3 relative to those in Im7 (Fig. 6c and d and Table 4) and consistent with the altered fluorescence properties of this species (Fig. 5).

Three residues were mutated in helix IV of Im7H3M3. Two residues (A77G and A78G) exhibit ϕ_I_ and ϕ_TS_ values similar to those of the corresponding residues of Im7 (Fig. 6g and h). Data obtained for the variant V69A at the N-terminus of helix IV indicate that this region of the protein does not make substantial stabilising contacts in the intermediate and transition states of wild-type Im7, as shown by the low ϕ-values of this variant. Destabilisation of native Im7H3M3 by this substitution, however, results in a ΔΔ*G*_UN_^o^ of < 2.5 kJ mol^−^ ^1^, a value too small to allow an accurate ϕ-value to be calculated for this site in the redesigned protein (Fig. 6g and h).^27^ Taken together, the data suggest that helix IV is at least partly formed in the intermediate ensemble and that the insertion of the extended helix III sequence into Im7 has only very minor effects on the structure of the intermediate ensemble, the rate-limiting transition-state ensemble and the native state in this region.

Mutation of residues in helix III in Im7H3M3 exhibit the largest differences in stability relative to substitutions in this region in wild-type Im7. The ϕ-values for residues mutated in helix III for Im7 suggest that the side chains of Thr51 and Ile54 make few stabilising contacts in the intermediate-state and transition-state ensembles (ϕ_I_ and ϕ_TS_ values for all residues are < 0.15; Table 4), despite destabilising the native state by ∼ 11 kJ mol^−^ ^1^ for I54V (Table 3 and Fig. 6e and f). However, the Im7H3M3 variant I54V shows a result very different from that of Im7 I54V, insofar as the native state is only marginally destabilised by this substitution (ΔΔ*G*_UN_^o^ ∼ 1.5 kJ mol^−^ ^1^ for I54V in Im7H3M3 compared with 11 kJ mol^−^ ^1^ for this substitution in wild-type Im7),^1^ while the stability of the intermediate ensemble is affected to approximately the same degree (ΔΔ*G*_UI_^o^ ∼ 2.1 kJ mol^− 1^) for both proteins (Table 4). The small ΔΔ*G*_UN_^o^ for this substitution in Im7H3M3 prevents the accurate determination of a ϕ-value,^27^ but the different effect of this mutation on the native-state stability of Im7H3M3 and Im7 is clear. Thus, the sequence differences between Im7 and Im7H3M3 appear to have negated the need for the side chain of Ile54 to be present in order to stabilise the native state.

In contrast with the Im7H3M3 I54V variant, the L53A variant of Im7H3M3 responds similarly to mutation as the L53A variant of Im7;^1^ in both cases, the truncation of residue 53 destabilises the native state to such an extent that the intermediate ensemble becomes the predominantly populated species at equilibrium. The observed rate constant of folding for the Im7H3M3 variant L53A is significantly faster (450 s^−^ ^1^) than that for Im7H3M3 (158 s^−^ ^1^), suggesting that the observed rate constant for the folding of the Im7H3M3 L53A variant monitors folding to the intermediate ensemble. In agreement with this, the folded state of the Im7H3M3 L53A variant in the absence of denaturant is more fluorescent than the denatured state and unfolds with an equilibrium free energy of 17.4 ± 2.0 kJ mol^−^ ^1^, consistent with the Δ*G*_UI_^o^ for Im7H3M3 (data not shown). Despite the fact that ϕ_I_ and ϕ_TS_ values could not be determined for the substitutions made in helix III of Im7H3M3, the similarity in the responses of the intermediate ensemble to mutations at residues 53 and 54 suggests that the presence of an extended helix III has not substantially altered the folding mechanism of Im7 in that the newly designed protein still folds to a stable native structure *via* a three-state transition, involving a hyperfluorescent intermediate with a three-helix core, as has been described for the wild-type protein.^1,2^

## Discussion

The data presented here indicate that the insertion of a polyalanine helix C-terminal to helix III in Im7 has a negligible effect on the folding mechanism of the protein. The variants designed to probe differences between the folding mechanisms of Im7 and Im7H3M3 exhibit similar ϕ-values for all of the residues examined (Fig. 6), consistent with the insertion of a large and highly helical sequence in place of helix III having little effect on the folding mechanism. Despite the wholesale similarities in ϕ-values, several residues in the natural helix III sequence displayed extensive differences in their response to mutation in the native state of the Im7H3M3 compared with Im7. The most striking difference observed was for the mutation I54V, which destabilises the native state of Im7 by ∼ 11 kJ mol^−^ ^1^ yet destabilises Im7H3M3 by only 1.5 kJ mol^−^ ^1^. Although surprising, this result is similar to that observed for the T4 lysozyme variant L133A, where the substitution had a large destabilising effect on the native state (ΔΔ*G*_UN_^o^ ∼ 20 kJ mol^−^ ^1^) but, when included as part of an 8-residue alanine helix, had a reduced destabilising effect on the native state (ΔΔ*G*_UN_^o^ ∼ 10 kJ mol^−^ ^1^). The results observed for the Im7H3M3 variant of Im7 suggest, therefore, that insertion of the polyalanine sequence allows compensation for the removal of the branched side chain of Ile54 such that the native protein is substantially less sensitive to mutation at this site, possibly by structural reorganisation of the core of the native structure upon truncation of the isoleucine side chain. As a consequence in the Im7H3M3 variant, the native state can still be preferentially populated despite truncation of this key residue in the native hydrophobic core.

The goal of this work was to determine whether helix III was the last helix to form during the folding of Im7 because it is the shortest of the four helices and has no significant helical propensity, or whether other features of the protein sequence dictate the formation of the three-helix intermediate en route to the native state. The data presented here indicate that the formation of a three-helix intermediate stabilised by both native and non-native interactions is an integral feature of the folding mechanism of Im7 and that folding *via* a three-state mechanism does not result from the short length and low helical propensity of the sequence of the native helix III. The results reveal that the helical propensity of helix III is not a key driver for the folding mechanism of Im7 and that formation of the full helix III requires docking onto the appropriate surface of the non-native three-helix bundle, where side-chain–side-chain interactions stabilise the sequence and allow the formation of helical structure. Thus, the three-helix bundle core can be considered as a folding template for helix III. The results add weight to the growing body of information on the folding mechanism of Im7, showing that folding *via* a three-helical intermediate is an obligate feature of the folding mechanism of this family of proteins. Indeed, a recent ϕ-value analysis of the folding of Im7, combined with molecular dynamics simulations, has provided insights into why a three-helical intermediate may be a ubiquitous feature of the rugged energy landscape of this family of proteins, in that functional residues located within helices II and III, particularly involving Tyr55, appear to form stabilising non-native contacts in the intermediate ensemble that occlude the binding site for helix III.^2^ The data presented here show that even when this region contains a preformed helical structure in the denatured state, helix III cannot compete with the rapid non-native docking of aromatic and hydrophobic residues early in Im7 folding such that a mechanism involving the canonical three-helical intermediate is preserved.

## Materials and Methods

### Mutagenesis and protein purification

Mutagenesis was carried out with the QuikChange site-directed mutagenesis kit (Stratagene) using the Im7 gene in pTrc99A as template.^1^ All proteins were expressed as His-tagged versions and were purified to homogeneity, as described previously.^1^ Expression and purification of isotopically labelled Im7H3M3 were carried out as described previously for Im7.^6^

### Im7H3M3 preparation

In order to construct an Im7H3M3 megaprimer, we performed PCR.^28^ Megaprimer PCR requires the use of three primers: two are non-mutagenic and contain either the NcoI restriction site or the HindIII restriction site that is found at the 5′ and 3′ ends of the Im7 gene, respectively, and the third is an internal mutagenic primer. Due to large-scale changes, a second extension primer was used in addition to the third internal mutagenic primer. In the first round of megaprimer PCR, the Im7 template DNA was used to produce the necessary extension product for the next round of PCR. The megaprimer was separated from the rest of the products of PCR by agarose gel electrophoresis and purified from the gel using the QIAquick Gel Extraction Kit (Qiagen, UK), in accordance with the manufacturer's instructions. In the second round, the newly extended Im7 template DNA was used in a second PCR to produce a further extended gene. A final, third round of PCR was performed, producing the required mutant gene, with short flanking sequences that contain relevant restriction sites for ligation into the expression vector pTrc99a. The full-length mutant gene was isolated by agarose gel electrophoresis and purified using a QIAquick Gel Extraction Kit. Pure mutated DNA was digested with appropriate restriction endonucleases and ligated into the plasmid pTrc99a. The DNA encoding the mutant gene was sequenced to ensure that it contained the desired change and no other.

### Analysis of peptide fragments of Im7 in TFE

Peptides were purchased from Peptide Protein Research (Fareham, UK) as 50% pure samples. Crude lyophilised peptide was purified to > 95% by reverse-phase HPLC using a C18 column (Dionex, USA). A stock peptide solution was prepared by dissolving 1.5 mg ml^−^ ^1^ of each peptide in buffer (50 mM sodium phosphate, pH 7.0), and its concentration was determined using the extinction coefficient.^29^ The sequence used to measure the helical propensity of helix II lacked a chromophore; therefore, Gly-Tyr was added to the C-terminus of the sequence to allow its concentration to be determined.^22^ Each peptide stock solution was then diluted 10-fold into a solution containing trifluoroethanol (0–80% volume TFE/total volume). All peptide concentrations were within the range 20–80 μM.^13^ In order to assess the effect of concentration on the peptide conformation, we made a second stock solution independently (concentration > 200 μM) and also measured it (data not shown). The molar ellipticity of all peptides was shown to be concentration independent over the range 20–200 μM. All peptides were soluble in the buffer used at all TFE concentrations. The samples were incubated overnight at 10 °C before analysis by CD (using a 1-mm pathlength cuvette; Hellma, UK). Sixteen scans were collected to improve the signal-to-noise ratio. All CD spectra were corrected for the signal of the buffer alone. Peptides equivalent to helices I, II and IV undergo a two-state transition between less helical forms and fully helical forms, as shown by the presence of an isodichroic point at 203 nm (Fig. 3). Consistent with this, analysis of the intensity of the signals at 190–195, 195–210 and 222 nm as a function of TFE concentration^30^ also indicated a two-state transition between coil and helix for these peptides (data not shown).

The observed ellipticity at 222 nm (θ_222_) in 0% TFE was used to determine the fraction helicity of each peptide using Eq. (1).^31,32^ In this analysis, the ellipticity at 222 nm is assumed to be linearly related to the mean helix content of a peptide:

(1) $${\text{f}}_{\text{H}}=\left(\theta_{222}-\theta_{\text{C}}\right)/\left(\theta_{\text{H}}-\theta_{\text{C}}\right)$$

where θ_C_ is the ellipticity of a random coil, and θ_H_ is the ellipticity of a fully formed helix. Both parameters are temperature dependent and derived from Eqs. (2) and (3), respectively:

(2) $$\theta_{\text{C}}=2,220-53\left(\text{temp}{}^{\circ}\text{C}\right)$$

(3) $$\theta_{\text{H}}=\left(\left(-44,000+250\left(\text{temp}{}^{\circ}\text{C}\right)\right)\left(1-3/{\text{N}}_{\text{r}}\right)\right)$$

where the values 2220 and − 44,000 cm^2^ deg dmol^−^ ^1^ peptide bond^−^ ^1^ are the ellipticities of the random coil and helix at 0 °C, respectively. The value − 44,000 cm^2^ deg dmol^−^ ^1^ peptide bond^−^ ^1^ is the ellipticity of an infinite length helix and, therefore, has a length correction factor included (1–3/*N*_r_), where *N*_r_ is the number of residues in the peptide chain.

### Kinetic data collection and analysis

All kinetic experiments were performed using an Applied Photophysics SX18.MV stopped-flow fluorimeter, as previously described.^1^ The temperature was maintained at 10 ± 0.1 °C using a Neslab-RTE300 circulating water bath. Fluorescence was excited at 280 nm with a 10-nm bandwidth, and a longpass filter was used to monitor the emission fluorescence at > 320 nm. Refolding experiments were performed by a 1:10 dilution of unfolded protein (initial concentration, 50 μM) in unfolding buffer (50 mM sodium phosphate, 0.4 M Na_2_SO_4_, 1 mM ethylenediaminetetraacetic acid and 8 M urea, pH 7.0) into a buffered urea solution to yield a final urea concentration within the range 0.75–7.75 M (in 0.25-M increments). Unfolding experiments were performed by a 1:10 dilution of protein (initial concentration, 50 μM) in refolding buffer (50 mM sodium phosphate, 0.4 M Na_2_SO_4_ and 1 mM ethylenediaminetetraacetic acid, pH 7.0) into a buffered urea solution to yield final urea concentrations within the range 3.0–7.75 M in 0.25-M increments. The resultant transients were fitted to a single exponential using the manufacturer's software (Spectrakinetic Workstation, v4.47; Applied Photophysics). The rate constants determined at each concentration were used to fit the resulting data to a three-state mechanism

(Scheme 1) $$U\overset{k_{\mathrm{UI}}}{\underset{k_{\mathrm{IU}}}{⇌}}I\overset{k_{\mathrm{IN}}}{\underset{k_{\mathrm{NI}}}{⇌}}N$$

where U, I and N are the unfolded, intermediate and native states, respectively, and *k_XY_* is the rate constant for folding between *X* and *Y*. Previous analysis of the folding of Im7 using ultrarapid mixing has shown the folding of the wild-type protein to be entirely consistent with such a model.^2,3^

The data for each protein measured were fitted using Igor 5.0 (Wavemetrics).^2^ When fitting data to Scheme 1, the formation of the intermediate was assumed to occur rapidly as a preequilibrium step. *k*_UI_ was fixed at 3000 s^−^ ^1^ for Im7 and all Im7H3M3 variants. The stability of the intermediate was then determined by allowing *k*_IU_ to vary. The macroscopic *M*-value *M*_UI_ was fixed at 4.7 kJ mol^−^ ^1^ M^−^ ^1^ (the value determined for the wild-type Im7H3M3) and assumed to be identical for each variant. The microscopic *m*-values *m*_IN_ and *m*_NI_ (*m*_*XY*_ represents the denaturant dependence of the natural logarithm of the rate constant *k_XY_*) were allowed to vary freely.

### ϕ-Value analysis

ϕ_I_ and ϕ_TS_ for the 24 variants of Im7H3M3 at pH 7.0 and 10 °C were determined by an analysis of folding and unfolding kinetics as a function of urea concentration. With only a single exception (L53A), folding was three state, as demonstrated by a kinetic rollover in the logarithm of the folding rate constant *versus* denaturant concentration at low denaturant concentration and by the presence of an increase in fluorescence signal in the dead time of the stopped-flow experiment (∼ 2.5 ms). Previous studies have shown that the only mechanism that can describe all the observed folding and unfolding data for Im7 is a three-state mechanism with an on-pathway intermediate.^2,3^ The chevron plots for Im7 and each variant, together with their initial and final fluorescence signals, were fitted to Scheme 1. It was assumed that the mutation altered the equilibrium stability of the intermediate and/or the folding/unfolding rate constants, while *M*_UI_ was assumed to be constant. Analysis of the kinetics of folding/unfolding showed no dependence on protein concentration (0.5–50 μM).

ϕ_TS_-values were calculated according to:

$$\Phi_{TS}=\frac{\Delta \Delta G_{U-TS}^{WT-VAR}}{\Delta \Delta G_{U-N}^{WT-VAR}}$$

where ΔΔ*G*_U–TS_^WT–VAR^ is the change in free energy of the transition state, referenced to the unfolded state, and is the difference in free energy between the native state and the unfolded state of the wild-type (WT) and variant (VAR) proteins, respectively.

Similarly, ϕ_I_ was calculated according to:

$$\Phi_{I}=\frac{\Delta \Delta G_{U-I}^{WT-VAR}}{\Delta \Delta G_{U-N}^{WT-VAR}}$$

Errors on all parameters were propagated mathematically.

### NMR spectroscopy

NMR spectra for the structure determination of Im7H3M3 were collected at 25 °C on Varian INOVA 500- and 600-MHz spectrometers at the University of East Anglia and on a Varian 900-MHz spectrometer at the Henry Wellcome Building NMR facility (University of Birmingham). A series of three-dimensional spectra [CBCA(CO)NH, HNCACB, HNCO, HCCH total correlated spectroscopy, H(CCO)NH, C(CO)NH, *^1^*H–*^1^*H–*^15^*N nuclear Overhauser enhancement spectroscopy (NOESY) heteronuclear single quantum coherence (HSQC) and *^1^*H–*^1^*H–*^13^*C NOESY-HSQC] was collected for complete assignments and nuclear Overhauser enhancement measurement. All NMR data were processed using NMRPipe^33^ and analysed using NMR View.^34^ The structure was determined with the software package ATNOS–CANDID–CYANA^35–37^ and refined in explicit solvent with Xplor-NIH^38,39^ from nuclear Overhauser enhancements obtained from *^1^*H–*^1^*H–*^13^*C and *^1^*H–*^1^*H–*^15^*N NOESY-HSQC spectra. None of the final structures contain violations of dihedral angle restraints larger than 10° or violations of distance restraints greater than 0.5 Å. For the final ensemble of 30 structures, the RMSD calculated over residues 2–94 is 0.68 ± 0.13 and 0.94 ± 0.09 Å for backbone and all heavy atoms, respectively. The Ramachandran plot for the ensemble of 30 structures determined with PROCHECK^40^ had 91.2% of residues in core regions, 8.4% of residues in allowed regions and 0.4% of residues in disallowed regions. Summary WHAT IF statistics^41^ are shown below. These statistics provide an overall summary of the quality of the structure as compared with current reliable structures. Structure *Z*-scores show a number of constraint-independent quality indicators. RMS *Z*-scores mostly give an impression of how well the model conforms to common refinement constraint values. The standard deviation shows variation over models in the ensemble, where appropriate.

Structure *Z*-scores: first-generation packing quality, − 0.167 ± 0.174; second-generation packing quality, − 0.772 ± 0.199; Ramachandran plot appearance, − 2.835 ± 0.416; χ^1^/χ^2^ rotamer normality, − 4.068 ± − 0.464; backbone conformation, − 1.464 ± 0.642.

RMS *Z*-scores: bond lengths, 1.178 ± − 0.009; bond angles, 0.555 ± 0.014; ω angle restraints, 0.115 ± 0.006; side-chain planarity, 0.945 ± 0.137; improper dihedral distribution, 0.897 ± − 0.090; inside/outside distribution, 0.988 ± 0.011.

### Accession numbers

Coordinates for the structure of Im7H3M3 have been deposited in the Protein Data Bank with accession number 2K0D.

## Figures and Tables

**Table 1:** Length and predicted average helical propensity for Im7 helices I, II, III and IV and the newly designed helix III of Im7H3M3 using the prediction algorithm AGADIR^12^

	Number of residues in helix	Percentage helicity	Measured percentage helicity
Helix I	13	7.8	14.1
Helix II	14	1.5	9.8
Helix III	6	0.2	0
Helix IV	14	3.1	11.2
Im7H3M3	13	14	16

Peptides corresponding to helices I, II, III and IV were constructed, and their α-helical content was determined using far-UV CD at 10 °C in 50 mM sodium phosphate (pH 7.0). The measured percent helicity for each peptide is also shown.

**Table 2:** Kinetic and thermodynamic properties of the folding/unfolding of wild-type Im7 and Im7H3M3

	*K*_UI_ (*M*_UI_)^a^	*K*_IN_ (*m*_IN_)	*K*_NI_ (*m*_NI_)	Δ*G*_UI_^o^^b^	Δ*G*_UN_^o^^c^
Im7	196.1 ± 166.7 (4.0 ± 0.1)	253.1 ± 5.6 (0.7 ± 0.0)	0.94 ± 0.1 (− 0.5 ± 0.0)	− 12.4 ± 0.1	− 25.6 ± 0.3
Im7H3M3	544.5 ± 92.9 (4.7 ± 0.2)	158.3 ± 4.1 (0.4 ± 0.1)	4.3 ± 0.3 (− 0.7 ± 0.0)	− 14.8 ± 0.4	− 23.3 ± 0.4

All rate constants are expressed in s^−^ ^1^, all *m*-values are expressed in kJ mol^−^ ^1^ M^−^ ^1^ and all free energies are expressed in kJ mol^−^ ^1^. Data were acquired at 10 °C in 50 mM sodium phosphate (pH 7.0) and 0.4 M Na_2_SO_4_.

a *M*_UI_ = (*m*_UI_ − *m*_IU_).

b Δ*G*_UI_^o^ = − *RT*ln(*K*_UI_).

c Δ*G*_UN_^o^ = − *RT*ln(*K*_UI_(*k*_IN_/*k*_NI_)).

**Table 3:** Kinetic and thermodynamic parameters for the folding/unfolding kinetics of Im7H3M3 variants

Protein	Location in native structure	*K*_UI_ (*M*_UI_)	*K*_IN_ (*m*_IN_)	*K*_NI_ (*m*_NI_)	Δ*G*_UI_^o^	Δ*G*_UN_^o^
Im7H3M3	—	544.5 ± 92.9 (4.7 ± 0.2)	158.3 ± 4.1 (0.4 ± 0.1)	4.26 ± 0.3 (− 0.7 ± 0.0)	− 14.8 ± 0.4	− 23.3 ± 0.4
A13G	Helix I (exposed)	155.4 ± 31.4 (4.7 ± 0.2)	174.6 ± 12.9 (0.5 ± 0.2)	2.4 ± 0.14 (− 0.9 ± 0.0)	− 11.9 ± 0.5	− 21.9 ± 0.5
V16A	Helix I (buried)	29.2 ± 5.2 (4.7 ± 0.2)	216 ± 8.8 (0.3 ± 0.1)	7.1 ± 0.19 (− 0.7 ± 0.0)	− 7.9 ± 0.4	− 15.9 ± 0.4
L19A	Helix I (buried)	24.7 ± 3.0 (4.7 ± 0.2)	259 ± 56.13 (1.8 ± 1.3)	24.5 ± 0.51 (− 1 ± 0.0)	− 7.5 ± 0.9	− 13.1 ± 0.9
K24A	Helix I (exposed)	333.3 ± 96.3 (4.7 ± 0.2)	142.3 ± 6.7 (0.2 ± 0.1)	5.99 ± 0.56 (− 0.7 ± 0.0)	− 13.7 ± 0.7	− 21.1 ± 0.7
A24G	Helix I (exposed)	368.1 ± 37.0 (4.7 ± 0.2)	157.1 ± 6.3 (0.4 ± 0.1)	7.89 ± 0.27 (− 0.7 ± 0.0)	− 13.9 ± 0.2	− 20.9 ± 0.3
V33A	Helix II (exposed)	874.6 ± 68.8 (4.7 ± 0.2)	143.8 ± 3.9 (0.6 ± 0.0)	4.36 ± 0.1 (− 0.9 ± 0.0)	− 15.9 ± 0.2	− 24.1 ± 0.2
D35A	Helix II (exposed)	3092.8 ± 223.2 (4.7 ± 0.2)	85.2 ± 2.19 (0.3 ± 0.03)	4.19 ± 0.28 (− 0.7 ± 0.0)	− 18.9 ± 0.2	− 25.9 ± 0.2
A35G	Helix II (exposed)	1818.2 ± 165.3 (4.7 ± 0.2)	99.6 ± 4.6 (0.17 ± 0.06)	7.5 ± 0.58 (− 0.6 ± 0.0)	− 17.6 ± 0.2	− 23.7 ± 0.3
L37A	Helix II (buried)	35.7 ± 10.5 (4.7 ± 0.2)	382.0 ± 27.4 (0.7 ± 0.3)	9.7 ± 0.4 (− 1.5 ± 0.0)	− 8.4 ± 0.7	− 17.0 ± 0.7
E39A	Helix II (exposed)	1734 ± 210 (4.7 ± 0.2)	134.7 ± 7.4 (0.55 ± 0.08)	8.79 ± 0.68 (− 0.5 ± 0.0)	− 17.5 ± 0.3	− 24.0 ± 0.4
A39G	Helix II (exposed)	1851 ± 171 (4.7 ± 0.2)	63.0 ± 2.3 (0.49 ± 0.05)	20.4 ± 0.66 (− 0.4 ± 0.0)	− 17.7 ± 0.2	− 20.4 ± 0.3
F41L	Helix II (buried)	170.5 ± 32.0 (4.7 ± 0.2)	421.7 ± 26.7 (0.1 ± 0.1)	10.3 ± 0.55 (− 0.7 ± 0.0)	− 12.1 ± 0.4	− 20.8 ± 0.5
V42A	Helix II (partially buried)	57.8 ± 17.8 (4.7 ± 0.2)	585.3 ± 41.8 (0.8 ± 0.2)	6.45 ± 0.68 (− 0.9 ± 0.0)	− 9.5 ± 0.7	− 20.1 ± 0.8
T51S	Helix III (buried)	290.1 ± 28.1 (4.7 ± 0.2)	204.2 ± 9.0 (0.5 ± 0.0)	4.62 ± 0.24 (− 1 ± 0.0)	− 13.4 ± 0.2	− 22.3 ± 0.3
A52G	Helix III (exposed)	288.5 ± 66.6 (4.7 ± 0.2)	177.5 ± 2.9 (0.5 ± 0.0)	5.3 ± 0.3 (− 0.9 ± 0.0)	− 13.3 ± 0.6	− 21.6 ± 0.6
L53A	Helix III (buried)^a^	163.9 ± 59.1 (4.7 ± 0.2)	−	−	− 17.4 ± 2.0	−
I54V	Helix III (buried)	222.2 ± 37.9 (4.7 ± 0.2)	225.7 ± 14.1 (0.5 ± 0.1)	4.80 ± 0.19 (− 1.3 ± 0.0)	− 12.7 ± 0.4	− 21.8 ± 0.4
AIIG	Helix III (exposed)	238.1 ± 30.2 (4.7 ± 0.2)	355 ± 15.1 (1.2 ± 0.1)	11.6 ± 0.28 (− 1 ± 0.0)	− 12.9 ± 0.3	− 20.9 ± 0.3
AVIG	Helix III (exposed)	137.0 ± 16.3 (4.7 ± 0.2)	221.4 ± 9.5 (0.3 ± 0.1)	5.9 ± 0.18 (− 0.7 ± 0.0)	− 11.6 ± 0.3	− 20.1 ± 0.3
V69A	Helix IV (buried)	219.0 ± 16.0 (4.7 ± 0.2)	192.6 ± 6.6 (0.3 ± 0.1)	5.2 ± 0.17 (− 0.9 ± 0.0)	− 12.7 ± 0.2	− 21.2 ± 0.2
K70A	Helix IV (exposed)	576.9 ± 36.6 (4.7 ± 0.2)	148.9 ± 4.6 (0.2 ± 0.05)	6.7 ± 0.3 (− 0.6 ± 0.0)	− 14.9 ± 0.2	− 22.3 ± 0.2
A70G	Helix IV (exposed)	87.5 ± 12.7 (4.7 ± 0.2)	240.3 ± 11.9 (0.7 ± 0.1)	6.0 ± 0.18 (− 0.8 ± 0.0)	− 10.5 ± 0.3	− 19.2 ± 0.4
A77G	Helix IV (exposed)	140.8 ± 23.1 (4.7 ± 0.2)	132. ± 7.2 (0.9 ± 0.1)	8.8 ± 0.28 (− 0.6 ± 0.0)	− 11.6 ± 0.4	− 18.0 ± 0.4
A78G	Helix IV (exposed)	91.7 ± 14.6 (4.7 ± 0.2)	123.3 ± 6.2 (0.4 ± 0.1)	9.2 ± 0.2 (− 0.7 ± 0.0)	− 10.6 ± 0.4	− 16.7 ± 0.4

All rate constants are expressed in s^−^ ^1^, all *m*-values are expressed in kJ mol^−^ ^1^ M^−^ ^1^ and all free energies are expressed in kJ mol^−^ ^1^. Data were acquired at 10 °C in 50 mM sodium phosphate (pH 7.0) and 0.4 M Na_2_SO_4_. Errors were calculated as described in Friel *et al.*^2^

a L53A folds predominantly (> 95%) to an intermediate state. The rates of folding of this variant *versus* the rates of folding of the denaturant were fitted to a two-state transition, yielding *k*_UI_ (450.7 ± 25.3 s^−^ ^1^) and *k*_IU_ (10.3 ± 0.7 s^−^ ^1^) from which Δ*G*_UI_^o^ (and *M*_UI_) values were calculated. Equilibrium studies confirmed that these parameters equate to those determined kinetically (Δ*G*_UI_^o^=− 17.4 ± 2.0 kJ mol^−^ ^1^ and *M*_UI_ = 4.7 ± 0.5 kJ mol^−^ ^1^ M^−^ ^1^, determined by equilibrium denaturation monitored by far-UV CD at 222 nm).

**Table 4:** ϕ-Values for Im7H3M3 variants and variants of wild-type Im7

	Im7H3M3 Φ_I_	Im7H3M3 Φ_TS_	Im7 Φ_I_	Im7 Φ_TS_
A13G	ND	ND	1.29 ± 0.13	1.25 ± 0.12
V16A	0.94 ± 0.03	0.84 ± 0.02	0.69 ± 0.03	0.77 ± 0.03
L19A	0.71 ± 0.09	0.60 ± 0.12	0.43 ± 0.08	0.34 ± 0.08
A35G	0.55 ± 0.09	0.39 ± 0.09	NA	NA
L37A	1.02 ± 0.00	0.69 ± 0.00	0.63 ± 0.08	0.51 ± 0.05
A39G	− 0.06 ± 0.10	0.48 ± 0.06	NA	NA
F41L	1.08 ± 0.12	0.16 ± 0.12	1.37 ± 0.09	0.25 ± 0.02
V42A	1.66 ± 0.20	0.69 ± 0.01	0.41 ± 0.05	0.13 ± 0.06
T51S	ND	ND	0.11 ± 0.05	0.09 ± 0.05
I54V	ND	ND	0.08 ± 0.03	0.12 ± 0.03
AVIG	1.0 ± 0.07	0.75 ± 0.06	−	−
V69A	ND	ND	0.18 ± 0.1	0.12 ± 0.11
A70G	1.45 ± 0.1	1.09 ± 0.05	NA	NA
A77G	0.60 ± 0.05	0.68 ± 0.04	0.81 ± 0.04	0.85 ± 0.04
A78G	0.64 ± 0.04	0.73 ± 0.03	0.61 ± 0.05	0.77 ± 0.04

Data for wild-type Im7 were taken from Capaldi *et al*.,^1^ with values recalculated from raw data as described by Friel *et al.*^2^ NA = data are not available since equivalent mutations were not created in the background of wild-type Im7. ND = not determined since ΔΔ*G*_UN_^o^ < 2.5 kJ mol^−^ ^1^.^27^
